# Effects of chemogenetic virus injection and clozapine administration in spinal cord injury

**DOI:** 10.1016/j.neurot.2025.e00547

**Published:** 2025-02-14

**Authors:** Ji Hyeon Kim, Sae Yeon Hwang, Hye-Lan Lee, Sol Lip Yoon, Yoon Ha, Hye Yeong Lee, Seungjun Ryu

**Affiliations:** a Spine & Spinal Cord Institute, Department of Neurosurgery, College of Medicine, Yonsei University, Seoul 03722, Republic of Korea; b POSTECH Biotech Center, Pohang University of Science and Technology (POSTECH), Pohang, Gyeongbuk, 37673, Republic of Korea; c Life Science Cluster, Center for Cognition and Sociality, Institute for Basic Science (IBS), Daejeon, Republic of Korea; d Department of Neurosurgery, School of Medicine, Eulji University, Daejeon, Republic of Korea

**Keywords:** AAV5-hSyn-hM3Dq-eYFP, Clozapine, Gq signaling pathway, Neuroregeneration, Designer receptors exclusively activated by designer drugs (DREADDs)

## Abstract

Neuromodulation therapy using chemogenetic stimulation has shown potential in enhancing motor recovery and neuroregeneration following spinal cord injury (SCI). These therapeutic benefits are hypothesized to result from the promotion of neuroplasticity, particularly when administered during the acute phase of injury. In this study, we investigated the effects of chemogenetic stimulation using Designer Receptors Exclusively Activated by Designer Drugs (DREADDs) in conjunction with clozapine, a ligand for receptor activation. DREADDs enable targeted, reversible neuromodulation, facilitating the histological characterization of engineered neurons. We utilized these receptors to modulate G-protein-coupled receptor (GPCR) signaling pathways, leading to the activation or inhibition of intracellular signaling. The objective was to determine whether the administration of DREADDs and clozapine (0.1 ​mg/kg) could enhance motor function and neuronal recovery, particularly when applied during the acute phase of SCI. Weekly behavioral assessments demonstrated significant improvements in motor skills and neuronal regeneration in treated animals compared to controls, with the most pronounced effects observed when stimulation was initiated early after injury. These enhancements in neuroplasticity were reflected in improved ladder rung test scores and Basso, Beattie, and Bresnahan (BBB) scale results in DREADDs-treated rats. Histological analyses, including immunohistochemistry (IHC) staining, Western blotting, and quantitative reverse transcription PCR (qRT-PCR), confirmed that the treatment group exhibited a higher density of neurons, increased signaling protein expression, and reduced inflammatory markers. These findings suggest that chemogenetic stimulation, particularly when administered during the acute phase, effectively promotes neuroregeneration and motor recovery. Future research should focus on assessing the long-term safety and efficacy of chemogenetic virus injection and clozapine administration, with an emphasis on the timing of intervention.

## Graphical abstract

**Figure:**
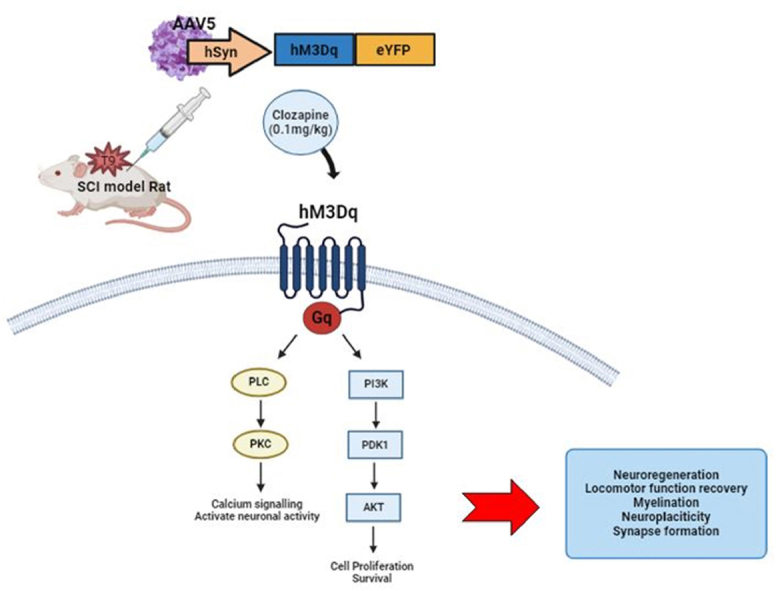

## Introduction

Spinal cord injury (SCI) is a devastating neurological condition that leads to significant motor, sensory, and autonomic dysfunction. The central nervous system (CNS), which includes the brain and spinal cord, faces unique challenges in regenerating following injury, with no current treatment capable of fully restoring pre-injury function [1,2]. Existing therapeutic approaches primarily focus on preventing further damage through surgical intervention and rehabilitation, with limited success in achieving substantial recovery. The pathophysiology of SCI is complex, involving acute and chronic phases characterized by ischemia, oxidative stress, inflammation, apoptosis, and extensive motor dysfunction [3]. Despite numerous efforts to mitigate these neurodegenerative processes and reduce secondary damage, the inherent complexity of CNS protective and healing mechanisms has made achieving significant functional recovery challenging [4,5].

Globally, the incidence of SCI is estimated to range from 250,000 to 500,000 cases annually, with current treatments offering mainly supportive care, leaving patients to cope with lifelong disabilities [6]. The intricate and variable outcomes following SCI contribute to the limited success of available treatments. The disruption and disorganization of interactions among key cell types—such as neurons, astrocytes, microglia, and oligodendrocytes—after SCI further complicate recovery efforts [7,8]. Although existing interventions, including pharmaceuticals, neuronal implants, and stem cell therapies, have shown some promise in improving neuroinflammation, axonal growth, and myelination, they often provide only short-term benefits and fail to fully counteract the detrimental effects of SCI [9].

A detailed understanding of SCI pathophysiology, particularly the cellular interactions and molecular pathways involved in both the acute and chronic phases, is crucial for developing effective therapeutic strategies [3]. Recent research suggests that interventions initiated during the acute phase of SCI may be more effective in promoting neuroregeneration and functional recovery than those applied during the chronic phase [10]. Following SCI, various molecules in the microenvironment, such as interleukin-1β (IL-1β), tumor necrosis factor-α (TNF-α), and damage-associated molecular patterns (DAMPs), activate multiple signaling pathways, including the mechanistic target of rapamycin (mTOR), extracellular signal-regulated kinase (ERK)/mitogen-activated protein kinase (MAPK), and phosphatidylinositol-3-kinase (PI3K)/serine-threonine kinase (Akt). These pathways play critical roles in neuroplasticity, inflammation, and apoptosis, influencing the progression of SCI and the extent of recovery [[11], [12], [13]].

Recent advancements in chemogenetic technology have introduced Designer Receptors Exclusively Activated by Designer Drugs (DREADDs) as a promising therapeutic approach for CNS disorders [14,15]. DREADDs offer a powerful chemical-genetic technique to remotely control neural activity and cell signaling [16,17]. Gq-coupled DREADDs (hM3Dq) enhance neural activity by activating ERK1/2 and increasing intracellular calcium, while Gi-coupled DREADDs (hM4Di) inhibit neural activity by reducing cyclic adenosine monophosphate (cAMP) levels and increasing G protein-coupled inwardly rectifying potassium channel currents [[18], [19], [20]]. The specificity of synthetic ligands for DREADDs and the receptors’ insensitivity to endogenous ligands have greatly expanded their experimental utility, making them valuable tools for investigating neural circuits and potential therapeutic interventions in SCI [21,22].

Clozapine, a metabolite of Clozapine-N-oxide (CNO), has emerged as an effective ligand for DREADDs, particularly due to its ability to cross the blood-brain barrier more efficiently than CNO [23]. While CNO has been the traditional ligand used in DREADDs studies, recent evidence suggests that clozapine binds DREADDs with higher affinity and more effectively activates the receptor, leading to enhanced neural activity and regeneration [22,24]. This study employs clozapine as the ligand for the AAV5-hSyn-hM3Dq-eYFP chemogenetic virus to promote neuronal activity and regeneration following SCI, with particular attention to the timing of intervention during the acute phase of injury [15,25].

In this study, we sought to elucidate the pathophysiological progression of SCI and assess the efficacy of chemogenetic stimulation in promoting neuroregeneration, focusing on the critical window during the acute phase of injury. By targeting the specific cellular and molecular pathways involved in SCI, our aim was to develop a therapeutic intervention that could enhance functional recovery and mitigate the devastating consequences of SCI.

## Materials and Methods

### Virus construction

The chemogenetic and control viruses used in this study were generated by the Korea Institute of Science and Technology (KIST, Seoul, Korea). The chemogenetic virus, AAV5-hSyn-hM3D(Gq)-eYFP, and the control virus, AAV5-hSyn-eYFP, were produced with specific concentrations of 5.9 ​× ​10ˆ13 ​GC/ml (#22-A107) for AAV5-hSyn-eYFP and 2.91 ​× ​10ˆ13 ​GC/ml (#22-A108) for AAV5-hSyn-hM3Dq-eYFP. These adeno-associated viral (AAV5) vectors were designed to exhibit central nervous system specificity, driven by the hSyn promoter, which is derived from the human synapsin 1 gene and ensures neuron-specific expression [22,26].

The hM3Dq receptor employed in the chemogenetic virus is a Designer Receptor Exclusively Activated by Designer Drugs (DREADDs) [27,28]. This receptor is a modified version of the human M3 muscarinic (hM3) receptor that is activated by the inert clozapine metabolite clozapine-N-oxide (CNO) [29,30]. Upon activation, the hM3Dq receptor initiates the Gq Signaling Pathway, leading to increased neuronal excitability [29,31]. Neurons expressing hM3Dq display significantly elevated firing rates when treated with clozapine, enabling precise control of neuronal activity for the purposes of this study.

### Spinal cord injury modeling and virus injection

Spinal cord injury (SCI) models were established using male Sprague-Dawley rats (200 ​± ​20 ​g; OrientBio, Gyeonggi-do, Korea). All procedures followed protocols approved by the Institutional Animal Care and Use Committee (IACUC; #2022–0085) and adhered to AAALAC guidelines. Anesthesia was induced using ketamine (100 ​mg/kg; Yuhan, Seoul, Korea), rompun (10 ​mg/kg; Bayer Korea, Seoul, Republic of Korea), and isoflurane (Hana, Seoul, Korea). A laminectomy was performed to expose the spinal cord, which was then compressed at the T9 level for 30 ​s using self-closing forceps (Fine Science Tools, North Vancouver, BC, Canada). This procedure resulted in lower-body paralysis. Following injury, the muscle layer was sutured, and the skin was closed with clips. Post-surgery, the rats were placed on a heating mat and administered cefazolin to prevent transduction. To ensure consistency and avoid including animals with incomplete injuries, rats with BBB scores exceeding 0–1 one day post-injury were excluded from the study.

Rats were divided into five groups: Sham, SCI, aSCI_Ctrl, aSCI_CS, and cSCI_CS. The Sham group consisted of untreated rats, while the SCI group included rats that received a 30-s compression injury at the T9 spinal cord segment. The aSCI_CS group was injected with chemogenetic virus 1 ​mm below the injury site 1 week after the injury. The aSCI_Ctrl group was injected with control virus 1 ​mm below the injury site 1 week after the injury. The cSCI_CS group was injected with chemogenetic virus 2 weeks after the injury [32]. The virus was injected stereotactically into the exact location. The virus was injected 0.7 ​mm to the right of the center of the spinal cord and 0.3 ​mm deep using a stereotactic frame [33]. Also Virus injections were performed using a 33-gauge microsyringe (Nanofil Syringe, 10 ​μL, NF33BV-2, WPI, USA), delivering 1 ​μL of virus at 0.1 ​μL/min. The needle was left in place for 10 ​min to allow viral diffusion, after which the incision was sutured, and the rats were placed on a heating mat for recovery. Cefazolin (25 ​mg/kg; Chong Kun Dang, Seoul, Korea) was administered for five days post-injection.

The aSCI_CS and cSCI_CS groups were compared to assess the effects of treatment timing on neuroregeneration. The pathophysiology of SCI involves two stages: the initial mechanical damage, followed by inflammation that activates resident and recruited immune cells, contributing to further tissue damage [34,35]. The cSCI_CS group was included to model the subacute stage of SCI, where delayed treatment is clinically relevant.

### Clozapine administration

Clozapine (0.1 ​mg/kg; 40-062379-01, Merck Sigma-Aldrich) was prepared as a stock solution at a concentration of 1 ​mg/ml by dissolving it in DMSO. For administration, this stock solution was diluted with distilled water (DW) to a final concentration of 100 ​μg/ml. The final injection dose was 100 ​μg/kg. Clozapine was administered intraperitoneally (i.p.). once daily until the animals were sacrificed at 8 weeks post-injury. To assess the immediate effects of clozapine on receptor activation and signaling processes, the final injection was given 30 ​min before the animals were sacrificed, after which the spinal cord was harvested for analysis.

### Behavioral analyses

Motor function recovery was assessed weekly with a particular focus on hindlimb-forelimb coordination. Two behavioral tests were utilized: the ladder rung test and the Basso, Beattie, Bresnahan (BBB) locomotor rating scale.

**Ladder Rung Test**: This test evaluates fine motor coordination by requiring rats to walk across a horizontal ladder with unevenly spaced rungs. The apparatus consisted of transparent acrylic side walls and a stabilizing metal bar beneath the ladder. The rungs were spaced at random intervals to prevent the rats from memorizing the pattern, thereby ensuring an accurate assessment of motor control. As the rats traversed the ladder, slips or placement errors of the hindlimbs were recorded. Two independent examiners scored each trial, and the percentage of correct steps (i.e., successful paw placement on the rungs without slipping) was calculated. The average percentage from multiple trials was used to quantify the rats' motor coordination and balance [36,37].

**Basso, Beattie, Bresnahan (BBB) Locomotor Rating Scale**: The BBB scale is a standard method for evaluating motor function following spinal cord injury [38]. Rats were placed in an open field and observed for spontaneous locomotion. Their gait was assessed on a 21-point scale, where 0 represented complete hindlimb paralysis (no observable movement), and 21 indicated normal locomotion with coordinated hindlimb-forelimb movement, consistent plantar stepping, and tail elevation. The evaluation criteria encompassed joint movement, weight-bearing capability, coordination, and tail movement during locomotion. Each rat's gait was scored by two independent examiners, and the final score was the average of these assessments, providing a comprehensive measure of motor recovery.

### Immunohistochemistry (IHC)

Spinal cord tissues were dissected and immediately fixed in 4 ​% paraformaldehyde (PFA) at 4 ​°C for one week. Following fixation, the tissues were transferred to 30 ​% sucrose for dehydration over an additional week. The dehydrated tissues were then embedded in OCT compound and stored at −80 ​°C until further processing. For immunohistochemistry, the frozen tissues were sectioned into 20 ​μm slices using a cryostat. The sections were air-dried at room temperature for 30 ​min and subsequently washed three times with cold 0.3 ​% Tween 20 (Sigma-Aldrich, P1379) in 1x PBS.

After washing, the sections were blocked at room temperature for 1 ​h using 10 ​% normal donkey serum in 0.3 ​% Triton X-100 (Sigma-Aldrich). The blocked sections were then incubated overnight at 4 ​°C with primary antibodies diluted in the blocking solution. The primary antibodies used in this study were rabbit anti-Tuj1 (1:1000; ab18207; Abcam), chicken anti-MAP2 (1:800; ab5392; Abcam), goat anti-GFAP (1:500; ab53554; Abcam), and chicken anti-MBP (1:400; ab134018; Abcam).

Following primary antibody incubation, the sections were washed three times with cold 0.3 ​% Tween 20 in 1x PBS. Species-specific secondary antibodies were then applied, including Alexa Fluor® 647 ​F(ab’)2 fragment donkey anti-rabbit IgG (H ​+ ​L) (1:500; 711-605-152; Jackson ImmunoResearch), Cy™3 donkey anti-chicken IgY (H ​+ ​L) (1:400; 703-165-155; Jackson ImmunoResearch), Alexa Fluor® 647 ​F(ab’)2 fragment donkey anti-goat IgG (H ​+ ​L) (1:250; 705-606-147; Jackson ImmunoResearch), and Cy™3 donkey anti-rabbit IgY (H ​+ ​L) (1:600; 711-165-152; Jackson ImmunoResearch). Secondary antibody incubation was carried out for 1 ​h at room temperature.

Following the secondary antibody treatment, the sections were washed three times with the washing buffer (0.3 ​% Tween 20 in 1x PBS). The nuclei were then stained using a DAPI-containing mounting solution (Vector Laboratories, Inc., Burlingame, CA, USA). Fluorescent images were acquired using a confocal laser microscope (LSM700, Carl Zeiss, Oberkochen, Germany) and subsequently processed using Image J software.

### Protein extraction and Western Blot analysis

For protein extraction, spinal cord tissues were harvested immediately following saline perfusion to remove blood. The tissues were placed in 1.5 ​ml tubes containing 1x PBS and flash-frozen in liquid nitrogen. The frozen tissues were then ground using a mortar and pestle in a liquid nitrogen-cooled container. To lyse the tissues, a modified Radio-Immunoprecipitation Assay (RIPA) buffer (#89901, Thermo Fisher, USA) supplemented with 1x phosphatase inhibitor (#3200001, GenDEPOT, USA) and 1x protease inhibitor cocktail (#78430, Thermo Fisher, USA) was added. The samples were kept on ice for 30 ​min, with vortexing every 3 ​min to ensure thorough lysis. After lysis, the samples were centrifuged at 13,200 ​rpm for 30 ​min at 4 ​°C, and the supernatant, containing the extracted proteins, was collected. The protein concentration was quantified using a Bicinchoninic Acid (BCA) protein assay (#23228, 23224, Thermo Fisher, USA), calibrated against a Bovine Serum Albumin (BSA) standard curve (0–2 ​mg/ml; #23209, Thermo Fisher, USA).

For Western blot analysis, equal amounts of protein (20 ​μg per lane) were separated by SDS-PAGE and transferred onto polyvinylidene difluoride (PVDF) membranes (#IPVH00010, Millipore, USA). The membranes were blocked with 1 ​% Bovine Serum Albumin (BSA) in TBS-T (Tris-buffered saline with Tween 20) for approximately 2 ​h at room temperature to prevent non-specific binding.

Following the blocking step, membranes were incubated overnight at 4 ​°C with primary antibodies diluted in 1 ​% BSA in TBS-T. The primary antibodies used included rabbit anti-Phospho-PLCβ (1:1000; #2821, Cell Signaling Technology), mouse anti-PLCβ (1:1000; sc-5291, Santa Cruz Biotechnology, Inc.), rabbit anti-Phospho-PKCγ (1:1000; ab5797, Abcam), rabbit anti-PKCγ (1:1000; ab71558, Abcam), rabbit anti-pAkt (1:1000; ab66138, Cell Signaling Technology), rabbit anti-AKT (1:1000; 9272S, Cell Signaling Technology), rabbit anti-BDNF (1:1000; ab108319, Abcam), rabbit anti-mGluR5 (1:1000; ab5675, Chemicon), rabbit anti-Neurocan (1:1000; ab279648, Abcam), rabbit anti-β-actin (1:5000; Abcam), and anti-PTPZ (1:1000; ab110479, Abcam).

After primary antibody incubation, the membranes were washed three times with 1x TBS-T for 5 ​min each and then incubated with species-specific HRP-conjugated secondary antibodies: goat anti-mouse IgG (1:2500; #32460, Invitrogen) and goat anti-rabbit IgG (1:2500; #32430, Invitrogen) for 2 ​h at room temperature. The membranes were then treated with an enhanced chemiluminescence (ECL) solution (#RPN2235, Cytiva, USA) to visualize the protein bands. The resulting signals were detected and analyzed using an imaging system, and densitometry analysis was performed using ImageJ software to quantify the relative protein expression levels across different experimental groups.

### RNA extraction and quantitative PCR analysis

For RNA extraction, spinal cord tissues were first perfused with saline to remove blood and then collected in 1.5 ​ml tubes containing RNAlater (Thermo Fisher, USA) to stabilize the RNA. The tissues were ground using a mortar and pestle in the presence of liquid nitrogen to maintain RNA integrity. Total RNA was extracted using TRIzol reagent (391307, Life Technologies, USA). After a 5-min incubation period, chloroform (#C2432, Sigma-Aldrich, USA) was added, and the mixture was gently inverted and incubated on ice for 3 ​min. The samples were then centrifuged at 13,200 ​rpm for 5 ​min at 4 ​°C. The aqueous phase containing RNA was carefully collected, and isopropyl alcohol (#858, Duksan, Korea) was added to precipitate the RNA, followed by storage at −20 ​°C for 1 ​h. After incubation, the samples were centrifuged again at 13,200 ​rpm for 5 ​min at 4 ​°C, the supernatant was discarded, and the RNA pellet was washed with 75 ​% ethanol. The RNA was then centrifuged once more at 13,200 ​rpm for 5 ​min at 4 ​°C, air-dried, and dissolved in 40 ​μL of diethyl pyrocarbonate (DEPC)-treated water. The RNA samples were stored at −80 ​°C until further use.

The concentration and purity of the extracted RNA were measured using a NanoDrop ND-1000 spectrophotometer (Thermo Fisher, USA). Complementary DNA (cDNA) was synthesized from the extracted RNA using the amfiRivert cDNA Synthesis Platinum Master Mix (#R5600, GenDEPOT, USA). Quantitative PCR (qPCR) was performed using Power SYBR® Green PCR Master Mix (#Q5603010, GenDEPOT, USA) in a Quantitative Real-Time PCR instrument (Applied Biosystems, Foster City, USA). The qPCR cycling conditions were as follows: initial denaturation at 95 ​°C for 3 ​min, followed by 40 cycles of denaturation at 95 ​°C for 5 ​s, annealing at 60 ​°C for 30 ​s, and extension at 95 ​°C for 15 ​s, with a final extension step at 60 ​°C for 1 ​min.

The amfiSure qGreen Q-PCR Master Mix (2X) (#Q5603010, GenDEPOT, USA) was used in conjunction with specific primers for the target genes: TNF-α, IL-10, iNOS, and CD206, with GAPDH serving as the reference gene. The sequences for the primers used were as follows:

TNF-α: Forward 5′-GTCAGAAAAGGAGATGCCCGA-3’; Reverse 5′-CCCAGGATATGGGGAAGCAC-3′

IL-10: Forward 5′-GAGTGAAGACCAGCAAAGGC-3’; Reverse 5′-CAACCCAAGTAACCCTTAAAGT-3′

iNOS: Forward 5′-ACCGAGATTGGAGTCCGAGA-3’; Reverse 5′-GCACAGCTGCATTGATCTCG-3′

CD206: Forward 5′-GTGCAAACACTGGGCAGAAG-3’; Reverse 5′-GCCTCCAGATCACTTGCTGT-3′

GAPDH: Forward 5′-CCTTCCGTGTTCCTACCCCC-3’; Reverse 5′-CCTGGTCCTCAGTGTAGCCC-3′

The relative gene expression levels were calculated and normalized using the comparative threshold cycle (Ct) method. The results were expressed as fold changes relative to control levels, calculated using the 2^ΔΔCt^ method.

### Eriochrome Cyanine (EC) staining

Eriochrome Cyanine (EC) staining was performed to assess myelin content in spinal cord tissue sections. Both longitudinal and cross-sections of the spinal cord were prepared and allowed to dry at room temperature for approximately 30 ​min. The dried sections were then immersed in acetone for 5 ​min to further fix the tissues, followed by an additional 15 ​min of air drying at room temperature.

The sections were subsequently stained with Eriochrome Cyanine solution (MERCK, Kenilworth, NJ, USA) for 30 ​min to visualize myelin. After staining, the sections were rinsed in distilled water for 5 ​min to remove excess dye. The stained sections were then treated with 5 ​% iron alum (221260, Sigma-Aldrich, USA) for 10 ​min to enhance color contrast, followed by another 5-min rinse in distilled water.

To prepare the tissue sections for microscopy, they were dehydrated through a graded ethanol series, consisting of sequential immersions in 70 ​%, 80 ​%, 90 ​%, and 100 ​% ethanol for 3 ​min each. Finally, the sections were cleared in xylene and mounted with a permanent mounting medium (Fisher Scientific, Hampton, NH, USA). The stained sections were allowed to dry for one day before being observed under a microscope for the assessment of myelin content.

### Statistical analysis

All data are presented as mean ​± ​standard error of the mean (SEM). Statistical analyses were performed to evaluate the significance of differences observed between experimental groups. Depending on the experimental design, either one-way or two-way analysis of variance (ANOVA) was employed for multiple group comparisons. Post-hoc analyses were conducted using Tukey's multiple comparisons test following one-way ANOVA, while a mixed-effects model was applied for two-way ANOVA. A p-value of less than 0.05 was considered statistically significant. Statistical significance levels were indicated as follows: p ​< ​0.05, ∗p ​< ​0.01, ∗∗p ​< ​0.001, and ∗∗∗p ​< ​0.0001.

GraphPad Prism 10.3 (GraphPad Software Inc., San Diego, CA, USA) was used for all statistical computations and graphical representations. Sample sizes varied across groups due to mortality during surgery or management, resulting in final group sizes of n ​= ​5 for the Sham group, n ​= ​14 for the SCI group, n ​= ​17 for the aSCI_Ctrl group, n ​= ​16 for the aSCI_CS group, and n ​= ​16 for the cSCI_CS group.

## Result

### Expression of chemogenetic and control viruses in neurons

**Fig. 1:**
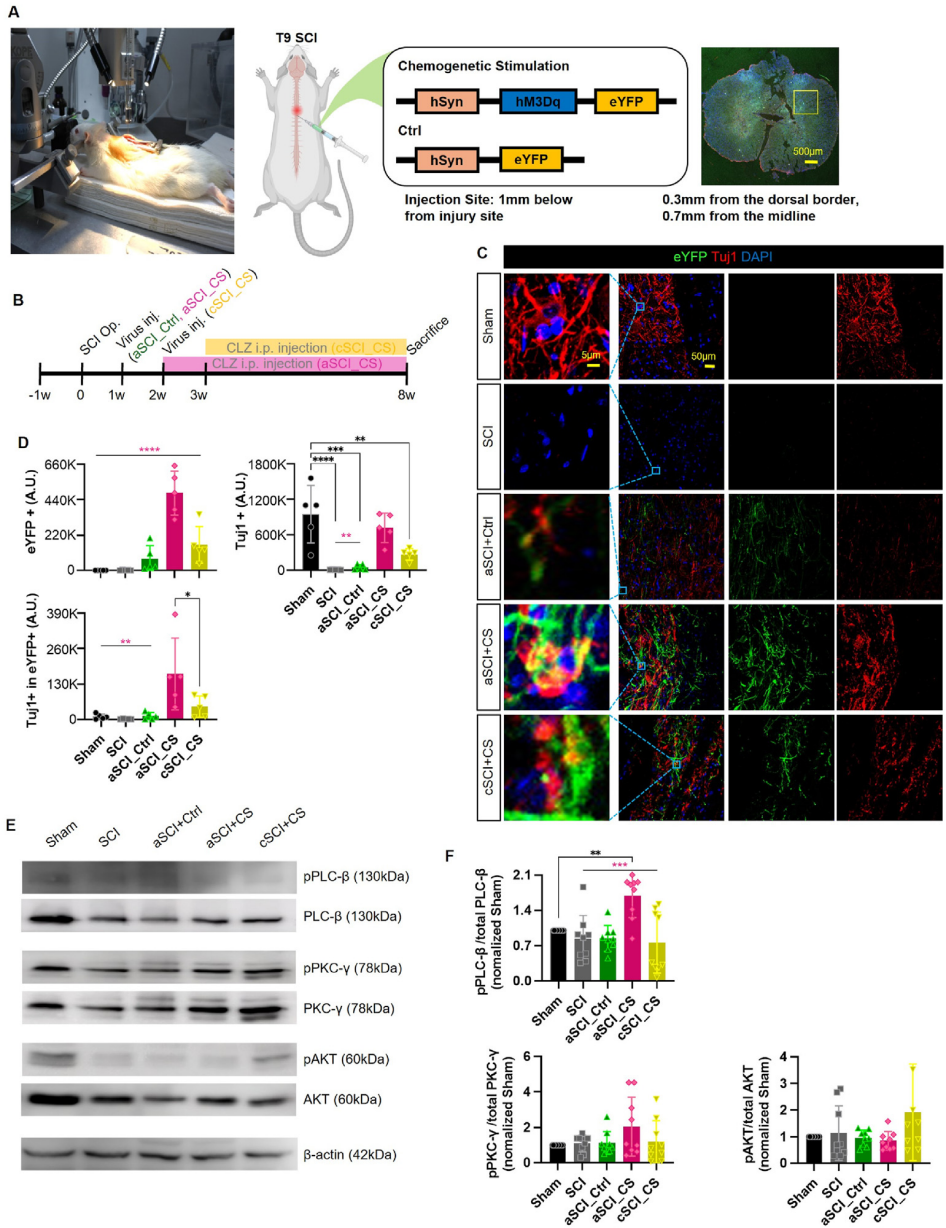
**Chemogenetic viruses and control viruses target Neurons and cause virus expression.** (A) Photograph of the surgical scene showing the stereotactic injection of the virus (depth 0.3 ​mm from the dorsal border, 0.7 ​mm from the midline) and an illustration indicating the spinal cord injury site and the virus injection location. The surgery was performed with precise injection using a stereotactic frame. Schematic illustration of the AAV5 vector hSyn-hM3Dq-eYFP containing the hM3Dq gene under the control of the human Syn1 promoter. On the right, cross-sectional confocal images show viral expression. (B) Schematic illustration of the time schedule for the in vivo experiments. (C) eYFP (Green), Tuj1 (Red), DAPI(Blue) IHC staining confocal images. The first image for each group is a 10× magnification of the box-marked area in the merge section. The scale bar shown 5 ​μm and 20 ​μm. (D) Quantification results of the intensity of eTFP^+^, Tuj1^+^ and Tuj1^+^ in eYFP using ImageJ, using images taken with a confocal microscope. (E) Western blot bands of phosphorylation of PLC-β, PLC-β, phosphorylation of PKC-γ, PKC-γ, phosphorylation of AKT, AKT. (F) Quantification results of Western blot bands using ImageJ. An equal amount of protein was loaded. Post-hoc analyses were conducted using Tukey's multiple comparisons test following one-way ANOVA. Statistical significance levels were indicated as follows: p ​< ​0.05, ∗p ​< ​0.01, ∗∗p ​< ​0.001, and ∗∗∗p ​< ​0.0001. Results are expressed as mean ​± ​SEM (∗∗*p* ​< ​0.01, ∗∗∗*p* ​< ​0.001 and ∗∗∗∗*p* ​< ​0.0001).

This study utilized an AAV5 vector to express the hM3Dq-eYFP gene under the control of the neuron-specific hSyn promoter. For control purposes, a separate AAV5 vector expressing eYFP alone was used (Fig. 1A). To confirm the targeted neuronal expression of the vector, the virus was injected into the lower spinal cord following injury. Expression of the viral vectors within neurons was confirmed by detecting the tagged eYFP, which emits green fluorescence and was visualized using fluorescence microscopy [39].

The experimental design included different timelines for each group. Following spinal cord injury (SCI), either the chemogenetic or control viruses were injected, followed by intraperitoneal injections of clozapine at a dosage of 0.1 ​mg/kg, which is known to be effective with minimal side effects. AAV5 mRNA expression [40,41]. The Sham group consisted of naïve rats that did not receive any virus or clozapine treatment. The SCI group included rats that sustained spinal cord injury without any subsequent treatment. The aSCI_Ctrl group received the control virus one week post-SCI, followed by clozapine injections beginning one week after the virus injection and continuing until the animals were sacrificed. The aSCI_CS group followed a similar timeline but received the chemogenetic virus instead. The cSCI_CS group received the chemogenetic virus two weeks post-SCI, with clozapine injections beginning one week later and continuing until sacrifice (Fig. 1B).

### Chemogenetic virus injection and clozapine administration promote neuronal activity

Clozapine, a DREADDs agonist, was administered intraperitoneally starting one week post-virus injection and continued daily until eight weeks before sacrifice. Additionally, a final dose of clozapine was given 30 ​min prior to sacrifice to evaluate the immediate effects of the chemogenetic virus and clozapine combination. Neuronal expression and recovery at the injury site were assessed using eYFP and Tuj1 markers (Fig. 1C). eYFP was used as a fluorescent marker for viral expression in neurons, while Tuj1 served as a marker for immature neurons. The co-localization of eYFP and Tuj1 (resulting in a yellow fluorescence) indicated the presence of immature neurons expressing the virus, suggesting ongoing neuronal activity and synaptic remodeling.

Quantification of these markers was performed using ImageJ software. The intensity of eYFP+ (arbitrary units, A.U.) reflected the levels of viral tansduction, Tuj1+ (A.U.) represented early-stage neurons, and Tuj1+ in eYFP+ (A.U.) indicated early-stage neurons within transduced cells. The quantification results (Fig. 1D) demonstrated increased expression of both eYFP and Tuj1 in the aSCI_CS and cSCI_CS groups, with the highest expression observed in the aSCI_CS group. These results suggest that chemogenic viruses effectively target existing neurons and activate neural activity, which may enhance neural firing and neuroplasticity, leading to improved behavioral outcomes [21]. In contrast, the cSCI_CS group exhibited lower expression levels of both markers compared to the aSCI_CS group, indicating a reduced level of viral expression and neuroregeneration in this group.

### Chemogenetic virus and clozapine administration engage the calcium signaling pathway

To investigate the involvement of the calcium signaling pathway in neuroregeneration, we analyzed key markers using Western blotting. The markers included phospholipase C-β (PLC-β), associated with neuroplasticity; protein kinase C-γ (PKC-γ), involved in gene expression and inflammatory responses; and protein kinase B (Akt), which plays a role in angiogenesis and stem cell regeneration [42]. Western blotting was performed on samples from three rats per group, using antibodies against phosphorylated forms of PLC- β (pPLC-β), PKC-γ (pPKC-γ), and AKT (pAKT), as well as their total forms, with β-actin serving as a loading control (Fig. 1E).

Quantitative analysis showed that the aSCI_CS group exhibited increased ratios of pPLC-β/total PLC-β and pPKC-γ/total PKC-γ, indicating higher activation of these signaling proteins. However, no significant differences were observed in the pAKT/total AKT ratios between the groups. By comparing the phosphorylated form to the total protein content, we assessed the proportion of activated protein, providing insights into the activity of the calcium signaling pathway and its role in nerve regeneration. A higher ratio suggests a greater level of protein activation, which is indicative of more active neuroregenerative processes [43].

The signaling pathway proceeds from PLC to PKC and then to AKT, with significant differences between groups observed primarily at the PLC level, which is directly downstream of the membrane receptor. PLC, as a membrane-associated signaling protein, is crucial for neuroplasticity and acts as a primary signal transducer [44]. Since PLC is positioned at the top of the signaling cascade, differences between groups are more pronounced at this level, with the signal diminishing as it progresses downstream. PKC, a downstream effector of PLC, is involved in gene expression, cell differentiation, and growth. While the aSCI_CS group displayed the highest quantitative values for PKC activation, these differences were not statistically significant across groups. Similarly, although the cSCI_CS group showed the highest values at the Akt level, no significant differences were observed between the groups [45,46].

The quantitative analysis revealed that chemogenetic virus injection and clozapine administration significantly influenced PLC activity, with the aSCI_CS group showing the highest PLC activation. This increased PLC activation was associated with enhanced neuroplasticity and correlated with the most substantial recovery observed in this group (Fig. 1F). These findings suggest that the viral vector encoding the chemogenetic receptor, hM3Dq effectively targeted neurons [47,48].

Further confirmation was obtained through immunohistochemistry (IHC) staining and confocal microscopy, which demonstrated that both the chemogenetic and control viruses were specifically expressed in neurons. The data also revealed that immature, early-stage neurons were regenerated within the populations expressing the viruses. Notably, the chemogenetic virus containing hM3Dq exhibited more than double the expression level compared to the control virus. This was corroborated by the Tuj1 quantitative analysis, which indicated the highest level of neuronal regeneration in the aSCI_CS group.

In summary, Western blot analysis confirmed that chemogenetic virus injection and clozapine administration significantly impacted signaling proteins involved in neuroplasticity. Groups that showed more pronounced regenerative effects exhibited higher expression levels of these signaling proteins, as evidenced by the quantitative analysis. However, no significant differences were found for signaling proteins located further downstream in the pathway. This suggests that proteins closer to membrane receptors and upstream in the signaling cascade receive stronger activation signals, with the signal diminishing as it progresses downstream, leading to reduced intergroup differences.

### Chemogenetic virus and clozapine administration enhance locomotor function recovery

**Fig. 2:**
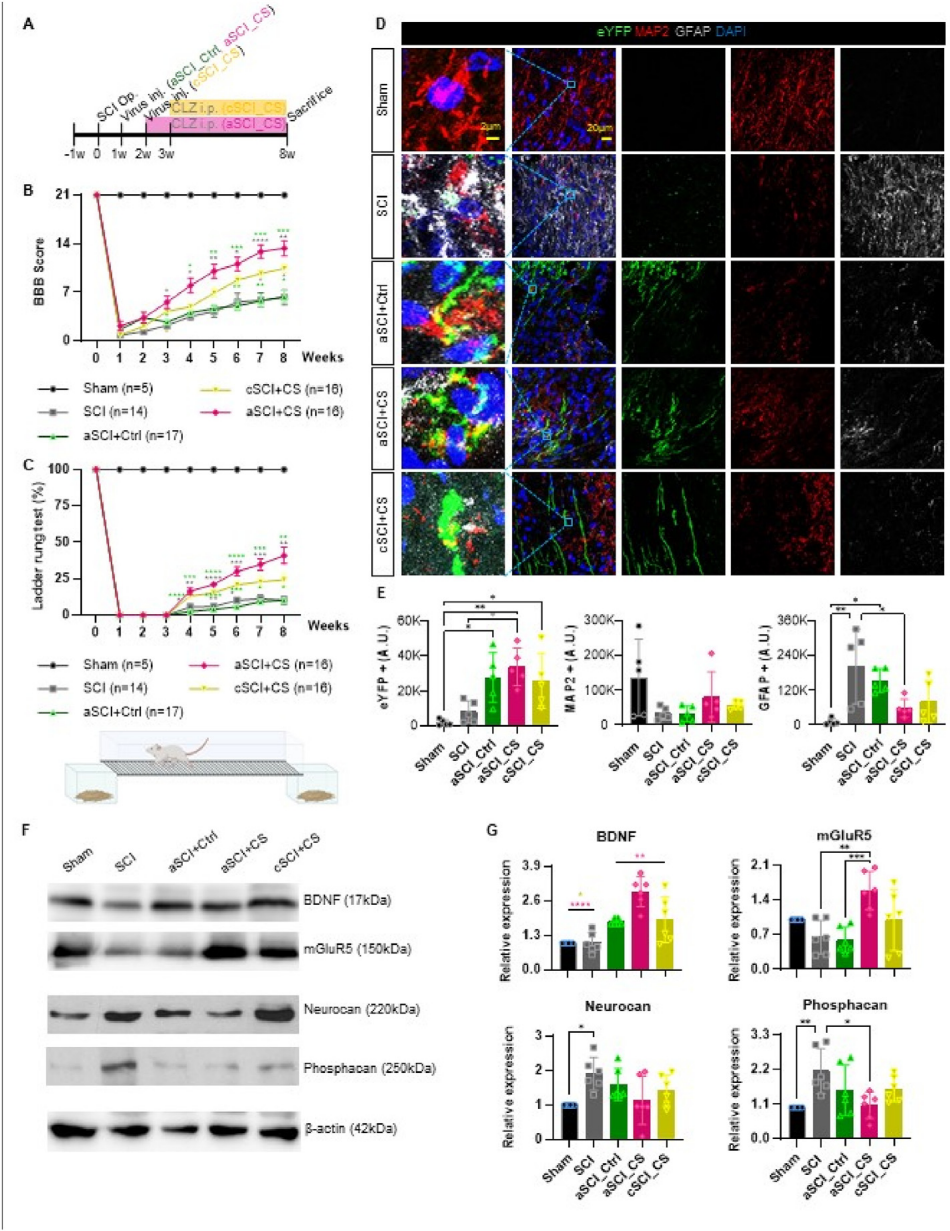
**Chemogenetic virus and clozapine promote functional regeneration and CSPGs interfere with neuroregeneration.** (A) Overall time schedule of the experiment (B) BBB scoring in all groups (Sham, SCI, aSCI_Ctrl, aSCI_CS, and cSCI_CS). A two-way ANOVA with Holm–Šídák's multiple comparisons was used for data analysis. (C) A graph showing the results of scoring the ladder-walking performance of all groups(Sham, SCI, aSCI_Ctrl, aSCI_CS, and cSCI_CS) and an image depicting the behavior of rats walking on the ladder. (D) eYFP (Green), MAP2 (Red), GFAP (Gray), DAPI(Blue) IHC staining confocal images. The first image for each group is a 10× magnification of the box-marked area in the merge section. The scale bars shown are 2 ​μm and 20 ​μm. (E) Quantification results of the intensity of eYFP^+^, MAP2^+,^ and GFAP^+^. (F) Western blot bands of BDNF, mGluR5, Neurocan, and Phosphacan. (G) Quantification results of Western blot bands using ImageJ. Post-hoc analyses were conducted using Tukey's multiple comparisons test following one-way ANOVA. An equal amount of protein was loaded. Results are expressed as mean ​± ​SEM (∗*p* ​< ​0.05, ∗∗*p* ​< ​0.01, ∗∗∗*p* ​< ​0.001, and ∗∗∗∗*p* ​< ​0.0001).

To evaluate the potential of the chemogenetic virus and clozapine combination to promote neuroregeneration in the spinal cord and restore locomotor function, we conducted two behavioral tests: the Basso, Beattie, Bresnahan (BBB) test and the Ladder Rung test (Fig. 2A). The BBB test, which scores rat gait on a scale from 0 to 21 (with higher scores indicating more normal gait), was used to assess overall motor function. The Ladder Rung test evaluated fine motor control and weight-bearing gait, where slips or dragging of the feet resulted in no score, and only successful, slip-free steps were counted [36].

In both tests, the aSCI_CS group exhibited the highest locomotor function scores. Significant improvements in motor recovery were observed in the aSCI_CS group compared to the aSCI_Ctrl and SCI groups, both of which demonstrated minimal recovery. Statistical analysis confirmed that the aSCI_CS group achieved the most substantial motor recovery in both behavioral tests (Fig. 2B–C).

### Chemogenetic virus and clozapine administration induce maturation of neurons

To assess neuronal regeneration and the potential inhibitory role of astrocytes, we performed immunohistochemical (IHC) staining using MAP2 (a marker of mature neurons) and GFAP (an astrocyte marker) (Fig. 2D). eYFP was used as a marker to confirm viral expression, with the highest expression observed in the aSCI_CS group, followed by the cSCI_CS and aSCI_Ctrl groups. In the Sham and SCI groups, which did not receive the virus, eYFP-positive cells were nearly absent. No eYFP-positive cells were observed in the Sham and SCI groups because eYFP expression depends on successful delivery of the viral vector and, without viral delivery, the eYFP gene would not be present in these groups and thus no fluorescence would be expected.

MAP2 expression was most pronounced in the aSCI_CS group, indicating that the AAV5-hSyn-hM3Dq-eYFP virus, in combination with clozapine, may have promoted neuronal activity and maturation. The merged images (Fig. 2D) display yellow regions where eYFP and MAP2 overlap, suggesting the presence of neurons in virus-transfected cells. Quantitative analysis showed that MAP2 expression was highest in the aSCI_CS group, supporting the notion that DREADD activation may enhance neuronal activity and synaptic remodeling in the injured spinal cord.

Conversely, GFAP expression was highest in the SCI group, indicating an increased presence of astrocytes, which are known to inhibit nerve cell regeneration. The aSCI_CS group, which exhibited the most significant functional recovery, showed the lowest GFAP expression, further supporting the notion that astrocyte proliferation was reduced in this group (Fig. 2E). The astrocyte is a key component in the formation of the early glial scar, which causes inflammation and inhibits nerve regeneration [49].

### Chemogenetic stimulation promotes regeneration and inhibits scar formation after spinal cord injury

To evaluate the regenerative effects of chemogenetic virus administration and clozapine treatment, we performed Western blot analysis using brain-derived neurotrophic factor (BDNF) and metabotropic glutamate receptor 5 (mGluR5) as markers of regeneration, and Neurocan and Phosphacan as markers of chondroitin sulfate proteoglycans (CSPGs), which are known to inhibit regeneration (Fig. 2F). CSPGs are critical inhibitors of neuronal growth and neuroplasticity following spinal cord injury (SCI), with increased levels correlating with reduced regenerative capacity. Neurocan and Phosphacan are specific CSPGs that appear at different stages post-injury and are key mediators of inflammatory microglial activation [50].

BDNF plays a crucial role in enhancing neural function, synaptic connections, promoting neuronal production, synaptic transmission, and plasticity within the central nervous system [51]. mGluR5, a member of the Gq protein-coupled glutamate receptor group 1 family, is involved in regulating neural activity and plasticity [52]. Western blot analysis revealed that BDNF and mGluR5 levels were highest in the aSCI_CS group, followed by the cSCI_CS group, indicating enhanced nerve regeneration in these groups. Conversely, the SCI and aSCI_Ctrl groups exhibited similar, lower levels of these markers, suggesting that clozapine alone does not promote regeneration in the absence of chemogenetic virus expression.

In contrast, Neurocan and Phosphacan levels were highest in the SCI group, which received only spinal cord injury without additional treatment. Neurocan is typically present for up to four weeks post-injury, after which its expression decreases. Phosphacan, however, begins to appear in around four weeks post-injury and continues to be expressed until approximately eight weeks. These distinct expression timelines make Neurocan and Phosphacan valuable markers for assessing glial scar formation and regenerative processes across different groups. The Western blot results confirmed that synaptic transmission, and plasticity markers (BDNF and mGluR5) were elevated in groups with significant regeneration, whereas inhibitory markers (Neurocan and Phosphacan) were more prominent in groups with limited synaptic transmission, and plasticity [53].

The quantitative analysis of BDNF expression revealed the highest levels in the aSCI_CS group, with significant differences compared to both the aSCI_Ctrl and cSCI_CS groups. Similarly, mGluR5 expression was highest in the aSCI_CS group, with significant differences observed between this group and the SCI and aSCI_Ctrl groups. In contrast, Neurocan and Phosphacan levels, which inhibit regeneration, were elevated in the SCI group, where regeneration was least effective. Neurocan expression was highest in the SCI group, and Phosphacan followed a similar trend, with a significant difference observed between the SCI and aSCI_CS groups (Fig. 2G). In the aSCI_CS group, where synaptic transmission was most pronounced, the levels of CSPG markers Neurocan and Phosphacan were the lowest. This suggests that the administration of chemogenetic virus and clozapine effectively inhibited the production of CSPGs and promoted synaptic transmission, plasticity and neuronal activity.

These findings demonstrate that chemogenetic stimulation, facilitated by the combined administration of chemogenetic virus and clozapine, effectively promotes motor function recovery and neuronal regeneration while simultaneously reducing glial scar formation at the injury site.

### Chemogenetic stimulation promotes myelination at the injury site

**Fig. 3:**
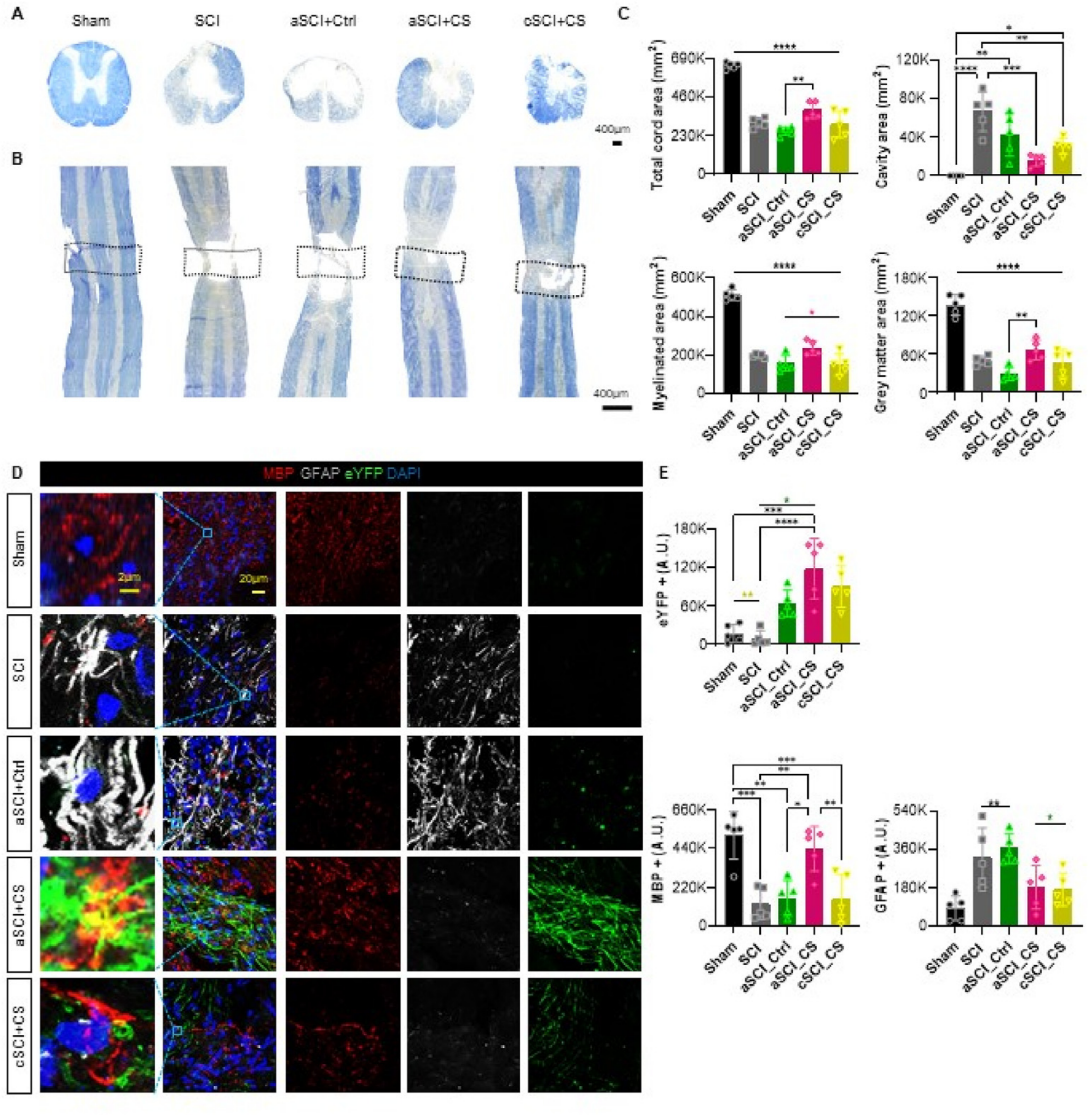
**Morphology of the spinal cord by group confirmed by EC staining and IHC staining and IHC analysis.** (A) Eriochrome cyanine staining of spinal cord cross-sections for myelin in all groups (sham, SCI, aSCI_Ctrl, aSCI_CS, cSCI_CS). Scale bar shown 400 ​μm. (B) Eriochrome cyanine staining of spinal cord longi section for myelin in all groups (sham, SCI, aSCI_Ctrl, aSCI_CS, cSCI_CS). Scale bar shown 400 ​μm. (C) The total cord area, cavity area, myelinated area, and gray matter area were quantified by image analysis. All data were processed using two-way analysis of variance with Tukey's multiple comparisons test. (D) MBP(Red), GFAP(Gray), and eYFP(Green), DAPI(Blue) IHC staining confocal images. The first image for each group is a 10× magnification of the box-marked area in the merge section. The scale bar shown 2 ​μm and 20 ​μm. (E) Quantification results of the intensity of eYFP^+^, MBP^+^ and GFAP^+^. Analysis was conducted using ordinary one-way ANOVA with Tukey's multiple comparisons test. The p-values are indicated as follows: ±SEM (∗*p* ​< ​0.05, ∗∗*p* ​< ​0.01, ∗∗∗*p* ​< ​0.001, and ∗∗∗∗*p* ​< ​0.0001).

To determine whether chemogenetic virus administration in combination with clozapine promotes myelination at the injury site, we assessed remyelination in the spinal cord using Eriochrome Cyanine (EC) staining on longitudinal and cross-sectional tissue sections (Fig. 3A–B). In EC staining, the cavity area represents the injury site, where a larger cavity area indicates more extensive damage and a smaller area suggests greater recovery. The results showed that the SCI group had the largest cavity area, reflecting the most significant injury, while the sham group, which did not undergo injury, had the smallest cavity area. Among the injured groups, the aSCI_CS group exhibited a reduced cavity area, indicating that remyelination occurred within the damaged site, contributing to the functional recovery of the nerve cells.

The myelinated area in the graph reflects the extent of nerve recovery, with increased myelination corresponding to greater regeneration and functional restoration. The aSCI_CS group displayed the highest myelination among the injured groups, confirming superior recovery at the injury site. Significant differences were observed between the aSCI_CS, aSCI_Ctrl, and cSCI_CS groups. The gray matter area, representing the region occupied by regenerated nerve cells within the cavity, was also evaluated. The spinal cord consists of gray matter (which contains nerve cells of the central nervous system) and white matter, with gray matter indicating remyelination in the damaged area. The aSCI_CS group demonstrated the most extensive remyelination, as evidenced by the largest myelinated area and gray matter coverage (Fig. 3C). In contrast, the SCI group, which only received the injury, exhibited the largest cavity area, indicating minimal recovery.

To further assess myelination, we utilized Myelin Basic Protein (MBP) and Glial Fibrillary Acidic Protein (GFAP) markers (Fig. 3D). MBP was used to detect proteins associated with myelination, with greater myelination resulting in higher MBP expression. Elevated levels of GFAP, on the other hand, indicate the presence of astrocytes, which are known to hinder regeneration. eYFP was employed as a marker to trace the expression of the injected viruses, as it is linked to the viral structure. Confocal microscopy images revealed that areas where MBP was present in eYFP-positive neurons appeared yellow, indicating enhanced myelination in the virus-injected cells. The highest MBP expression was observed in the sham group, followed by the aSCI_CS group. GFAP expression was reduced in both the aSCI_CS and cSCI_CS groups (Fig. 3E).

These results confirm that the aSCI_CS group experienced the most significant remyelination, indicating the highest treatment efficacy, consistent with the findings from previous analyses.

### Chemogenetic virus and clozapine administration influence inflammation and neuronal regeneration

**Fig. 4:**
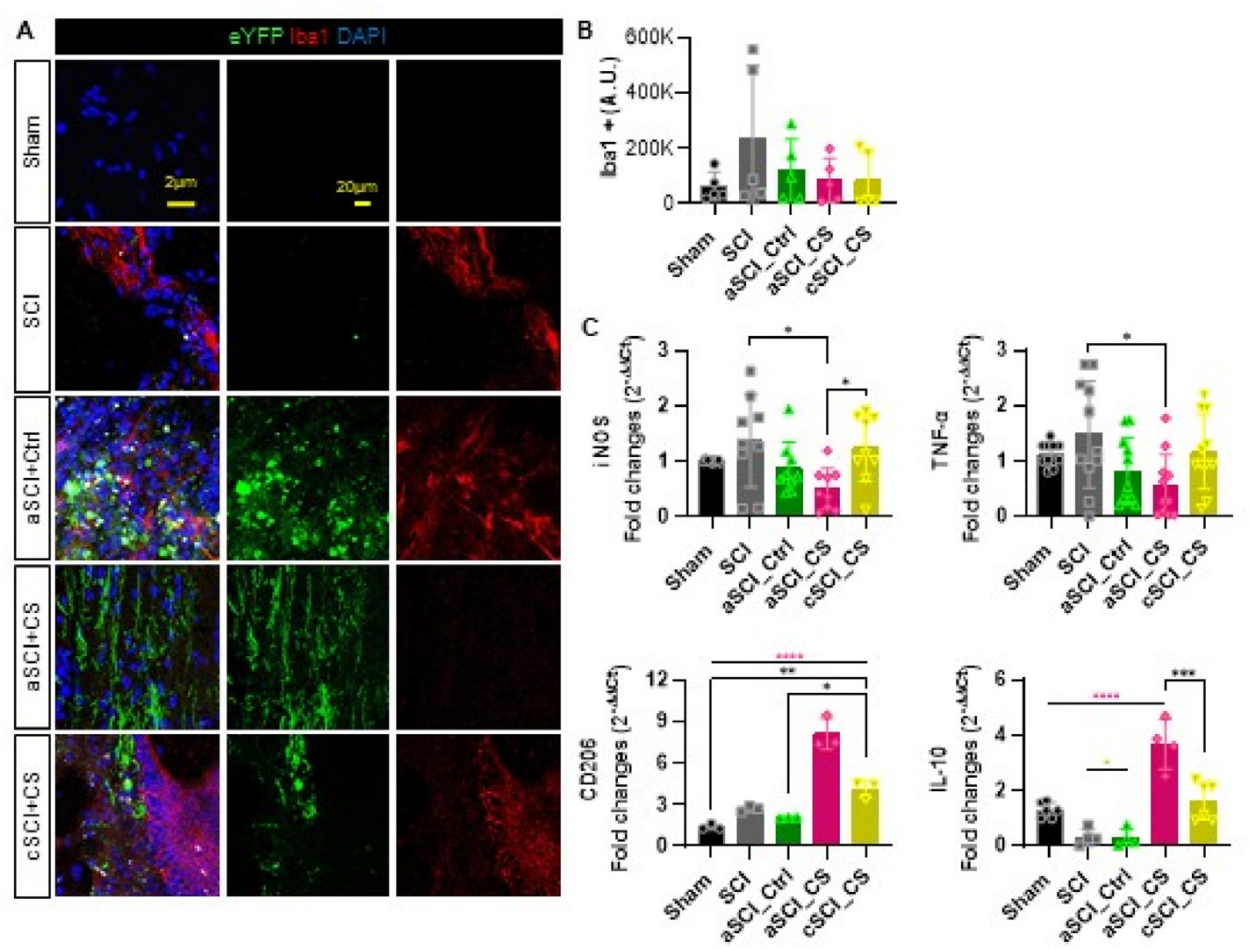
**Chemogeneic virus injection and clozapine administration promote anti-inflamatory effects.** (A) eYFP (Green), Iba1 (Red), DAPI(Blue) IHC staining confocal images. The scale bar shown 20 ​μm. (B) Quantification results of the intensity of Iba1^+^. (C) Quantification results of qRT-PCR values for macrophage markers (iNOS, TNF-α) and inflammatory markers(CD206 and IL-10). Results are expressed as mean ​± ​SEM (∗*p* ​< ​0.05, ∗∗*p* ​< ​0.01, ∗∗∗*p* ​< ​0.001, and ∗∗∗∗*p* ​< ​0.0001).

We investigated the anti-inflammatory effects of chemogenetic virus administration in combination with clozapine by assessing neuroinflammation and its impact on neuronal regeneration. Immunohistochemical (IHC) staining for Iba1, a microglia marker, was performed to monitor inflammatory responses at the injury site (Fig. 4A). Microglia play a critical role in neuroinflammation, and the Iba1 marker serves as an effective indicator of inflammatory activity. The results showed that the SCI group exhibited significantly higher levels of inflammation compared to the aSCI_Ctrl, aSCI_CS, and cSCI_CS groups. Since neither the SCI group nor the aSCI_Ctrl group received chemogenetic virus injections, the inflammatory response at the injury site remained elevated, as indicated by the high Iba1 expression. In contrast, both the aSCI_CS and cSCI_CS groups demonstrated a significant reduction in inflammation, suggesting that the combination of chemogenetic virus and clozapine synergistically reduced the inflammatory response.

To further explore the effects on inflammation, we employed quantitative reverse transcription PCR (qRT-PCR) to assess the expression levels of macrophage markers and inflammatory cytokines, specifically iNOS (an M1 marker), CD206 (an M2 marker), TNF-α (a pro-inflammatory cytokine), and IL-10 (an anti-inflammatory cytokine). The fold changes in these markers were measured across different groups (Fig. 4C). The SCI group exhibited higher fold changes in iNOS and TNF-α compared to the aSCI_CS group, indicating a heightened pro-inflammatory response with increased tissue damage and inflammation in the absence of chemogenetic intervention. The cSCI_CS group also showed elevated levels of these pro-inflammatory markers, which may be related to the timing of virus injection. The differences in quantitative values between the aSCI_CS and cSCI_CS groups suggest that earlier virus injection correlates with a greater nerve recovery effect.

Conversely, the fold changes for CD206 and IL-10 were highest in the aSCI_CS group, reflecting elevated anti-inflammatory activity. In contrast, the SCI and aSCI_Ctrl groups, which experienced the most damage and were untreated, exhibited higher pro-inflammatory activity with minimal anti-inflammatory response.

In summary, these findings demonstrate that chemogenetic virus and clozapine therapy significantly influence inflammatory activity. The groups with greater recovery, such as the aSCI_CS group, exhibited a reduced M1 response and lower pro-inflammatory activity, along with an enhanced M2 response and higher anti-inflammatory activity. This shift in the inflammatory profile is associated with increased nerve cell regeneration and improved recovery outcomes.

## Discussion

Chemogenetic stimulation using AAV5-hSyn-hM3Dq-eYFP in combination with clozapine significantly enhances neuroregeneration and motor recovery following spinal cord injury (SCI). Our comprehensive analysis, which included immunohistochemistry (IHC) staining, behavioral assessments, Western blotting, and quantitative real-time PCR (qRT-PCR), demonstrates that this therapeutic approach not only promotes neuronal survival and remyelination but also leads to functional improvements [54,55]. These findings underscore the potential of chemogenetic stimulation to facilitate neuroplasticity and recovery in an SCI model.

A key observation in this study is the markedly enhanced efficacy of chemogenetic stimulation when administered during the acute phase of SCI, as opposed to the chronic phase. Early intervention appears to leverage the body's intrinsic repair mechanisms more effectively, resulting in a more pronounced neuroregenerative response [10,56]. This highlights the critical importance of timely therapeutic intervention, as early treatment is likely to coincide with periods of heightened neuroplasticity and cellular responsiveness, thereby optimizing recovery outcomes [57,58].

The importance of early intervention is further corroborated by comparative analyses between acute and chronic phase treatments. Animals treated with chemogenetic stimulation during the acute phase exhibited significantly better motor function and neuroregeneration outcomes compared to those treated in the chronic phase. Behavioral assessments, including the Basso, Beattie, and Bresnahan (BBB) scale and the ladder rung test, revealed greater improvements in motor skills in the acute-phase group [59]. These behavioral outcomes were supported by histological data showing higher neuronal density, increased expression of regeneration-associated proteins, and reduced inflammatory markers. These results suggest that early intervention not only maximizes the regenerative potential of the injured spinal cord but also mitigates the negative consequences associated with delayed treatment [60,61].

The AAV5 vector used in this study has been shown to achieve 30–50 ​% transgene expression within one week of injection, with peak expression typically occurring by 2–3 weeks [62]. Given that our protocol required confirmation of spinal cord injury paralysis up to two weeks post-injury, viral injections and stimulation in the acute recovery group were performed one week after injury. This aligns with the early onset of AAV5 expression and the critical importance of early intervention during the acute phase of spinal cord recovery, which significantly impacts long-term outcomes. While maximal expression may not occur during the early acute phase, the ramping up of viral expression likely contributed to the therapeutic effects observed during the late acute and subacute phases [63].

The biological mechanisms underpinning the efficacy of early chemogenetic stimulation involve several critical pathways [56]. Activation of the hM3Dq receptor by clozapine initiates the Gq-signaling pathway, leading to increased intracellular Ca2+ levels, a key factor in promoting neuroplasticity and neuronal regeneration [57,64]. The elevation in Ca2+ levels enhances neuronal excitation and supports regeneration by facilitating neuronal interactions [65]. Western blot analyses confirmed that early chemogenetic stimulation upregulated proteins associated with neuronal regeneration and increased intracellular Ca2+ concentrations in virus-transducred tissues. Moreover, brain-derived neurotrophic factor (BDNF) and metabotropic glutamate receptor 5 (mGluR5) were found to be upregulated following clozapine treatment in hM3Dq-expressing neurons, contributing to neuronal survival, synaptic plasticity, and regeneration [[66], [67], [68]]. That is, the hm3Dq receptor binds to the clozapine ligand and activates Gq signaling, which increases calcium concentration, increases neural activity, and induces neural regeneration. PKC and PLC promote calcium signaling and neural activity. Gq signaling ultimately activates PLC, which induces PKC activation and promotes neural activity and neuroplasticity [69].

Additionally, early intervention was associated with a reduction in inhibitory factors such as chondroitin sulfate proteoglycans (CSPGs), which are known to impede axonal growth and neuroplasticity [70]. The suppression of these inhibitory molecules during the acute phase is particularly noteworthy, as it facilitates the regeneration of damaged neurons and contributes to the observed improvements in motor function [71]. The role of phospholipase C-β (PLC-β) in this process was also highlighted, with significant differences in its expression observed, particularly in the acute-phase treatment group, indicating enhanced nerve regeneration [72,73].

The comparative effectiveness of chemogenetic stimulation between acute and chronic phases is evident in the differential outcomes observed across treatment groups. The acute-phase group demonstrated superior motor recovery and higher levels of neuroregeneration, as indicated by increased expression of regeneration-associated proteins, reduced inflammatory markers, and improved behavioral performance [47]. In contrast, the chronic-phase group exhibited less pronounced improvements, suggesting that delayed intervention may miss the critical window for maximizing the neuroregenerative response [74]. These findings emphasize the necessity of early therapeutic application to achieve optimal outcomes.

The results of this study hold significant implications for the clinical management of SCI. The success of early chemogenetic stimulation suggests that therapeutic protocols should prioritize early intervention to maximize neuroregenerative outcomes. Integrating this approach into existing treatment strategies for SCI could offer a novel avenue for enhancing patient recovery [75]. The use of clozapine as a ligand for DREADDs, given its established pharmacokinetics and safety profile at low doses, presents a viable option for clinical application, though careful dosing is required to minimize off-target effects [23]. To minimize off-target effects, we administered the lowest possible dose of clozapine, and in our experiments, no behavioral effects such as limb movements or muscle tone after ligand administration were observed. This suggests that nonspecific binding was minimized.

During our study, we carefully monitored animals after clozapine administration to identify potential “off-target” effects, such as unintended limb movements or muscle twitches. No such effects were observed, suggesting that the effects of DREADD activation were localized to targeted neuronal populations. While it is possible that alpha-motoneurons or interneurons synapsing onto alpha-motoneurons could be transduced, our findings indicate that this did not result in detectable activation of muscle groups or unintended behavioral outcomes. These observations further support the specificity of this approach and highlight the importance of precise experimental design in chemogenetic studies.

While clozapine was selected for its ability to cross the blood-brain barrier and effectively activate DREADDs, its use is not without potential off-target effects [22]. Clozapine's actions on other CNS receptors, including those for dopamine, serotonin, norepinephrine, and histamine, could influence recovery, particularly in the chronic phase where receptor sensitivity and expression may differ [76]. For example, off-target activation of serotonin receptors might modulate mood and affective states, potentially impacting behavioral outcomes [22]. Additionally, chronic activation of dopamine receptors could alter motor function, complicating the assessment of recovery. Thus, while clozapine is an effective ligand for DREADDs in this context, its broad pharmacological profile necessitates careful consideration of dosing and potential side effects [23,30]. Future research should focus on assessing the long-term neuroprotective effects and safety of chemogenetic stimulation, particularly concerning the extended use of antiviral therapies and clozapine. Given clozapine's complex pharmacology, which affects multiple CNS receptors, it is crucial to explore the long-term implications of its use, especially at low doses [77,78]. Moreover, investigating the potential for translating these findings to other neurological conditions, such as traumatic brain injury or stroke, where timely intervention could similarly enhance outcomes, is essential [79]. Future studies should also determine optimal timing and dosing regimens for effectively implementing chemogenetic stimulation as a standard treatment for SCI and potentially other neurodegenerative diseases [3].

The potential for adapting chemogenetic stimulation for other neurological conditions, such as traumatic brain injury or stroke, is significant. Although the pathology of these conditions differs from that of SCI, the fundamental principles of enhancing neuroplasticity and reducing inhibitory factors remain applicable [80]. Future research should investigate how chemogenetic stimulation might be tailored to the specific pathophysiological features of these disorders, with a particular emphasis on early intervention to optimize therapeutic outcomes [1]. This may involve adapting the timing, dosing, and target pathways to meet the unique needs of each condition, thereby broadening the application of chemogenetic tools in neuroregenerative medicine [75,81].

Our study demonstrates that chemogenetic stimulation using the AAV5-hSyn-hM3Dq-eYFP virus, in combination with clozapine, significantly enhances neuroregeneration and remyelination following spinal cord injury (SCI). This therapeutic approach effectively activates the calcium signaling pathway, improves locomotor function recovery, promotes neuronal maturation, and reduces both inflammation and scar formation. These findings suggest that chemogenetic stimulation could be a promising strategy for SCI treatment.

Moreover, our results indicate that the post-SCI administration of the chemogenetic virus (hM3Dq), followed by sustained clozapine stimulation, facilitates early expression of neuron-related genes and proteins, preservation of host spinal cord tissue, and substantial motor recovery. This highlights the potential of combining chemogenetic tools with clozapine to advance neural regeneration and functional recovery in SCI and possibly other neurological conditions.

## Author contributions

J.H.K., S.Y.H., and H.Y.L. contributed to investigation. J.H.K. also performed validation and contributed to writing the original draft. S.L.Y. contributed to investigation. H.Y.L. curated the data, performed formal analysis, validation, and visualization, and contributed to both writing the original draft and review & editing. Y.H. supervised the project and handled project administration. S.R. conceived the project, developed the methodology, acquired funding, supervised the project, and handled project administration.

## Ethics approval statements

All rat care and surgeries were performed in accordance with Association for Assessment and Accreditation of Laboratory Animal Care (AAALAC) regulations and were managed and supervised with Institutional Animal Care and Use Committee at Yonsei university college of medicine (IACUC; protocol number: 2022–0085) approval.

## Availability of data and materials

The drugs used in the study can be purchased at Sigma-Aldrich now. In addition, the data that support the findings of this study are available from the corresponding authors upon request.

## Declaration of competing interest

The authors declare the following financial interests/personal relationships which may be considered as potential competing interests: Seungjun Ryu reports financial support was provided by National Research Foundation of Korea(NRF). If there are other authors, they declare that they have no known competing financial interests or personal relationships that could have appeared to influence the work reported in this paper.
